# Development of a tool for coding safety-netting behaviours in primary care: a mixed-methods study using existing UK consultation recordings

**DOI:** 10.3399/bjgp19X706589

**Published:** 2019-11-19

**Authors:** Peter J Edwards, Matthew J Ridd, Emily Sanderson, Rebecca K Barnes

**Affiliations:** Centre for Academic Primary Care, Bristol Medical School, Population Health Sciences, University of Bristol, Bristol.; Centre for Academic Primary Care, Bristol Medical School, Population Health Sciences, University of Bristol, Bristol.; Centre for Academic Primary Care, Bristol Medical School, Population Health Sciences, University of Bristol, Bristol.; Centre for Academic Primary Care, Bristol Medical School, Population Health Sciences, University of Bristol, Bristol.

**Keywords:** clinical coding, health communication, patient safety, primary health care, reproducibility of results, safety netting, video recording

## Abstract

**Background:**

Safety netting is recommended in a variety of clinical settings, yet there are no tools to record clinician safety-netting communication behaviours.

**Aim:**

To develop and assess the inter-rater reliability (IRR) of a coding tool designed to assess safety-netting communication behaviours in primary care consultations.

**Design and setting:**

A mixed-methods study using an existing dataset of video-and audio-recorded UK primary care consultations.

**Method:**

Key components that should be assessed in a coding tool were identified using the published literature and relevant guidelines. An iterative approach was utilised to continuously refine and generate new codes based on the application to real-life consultations. After the codebook had been generated, it was applied to 35 problems in 24 consultations independently by two coders. IRR scores were then calculated.

**Results:**

The tool allows for the identification and quantification of the key elements of safety-netting advice including: who initiates the advice and at which stage of the consultation; the number of symptoms or conditions the patient is advised to look out for; what action patients should take and how urgently; as well as capturing how patients respond to such advice plus important contextual codes such as the communication of diagnostic uncertainty, the expected time course of an illness, and any follow-up plans. The final tool had substantial levels of IRR with the mean average agreement for the final tool being 88% (*κ =* 0.66).

**Conclusion:**

The authors have developed a novel tool that can reliably code the extent of clinician safety-netting communication behaviours.

## INTRODUCTION

‘Safety netting’, also known as ‘contingency planning’, has become an integral part of clinical care in a variety of settings and is now widely considered to form part of best practice in primary care.^1^^–^3 Despite this, there is a lack of evidence on how often and which safety-netting strategies are utilised in clinical practice; how patients immediately respond; and the effects of safety netting on patient safety.^4^^,^^5^

Neighbour initially described the safety-netting checkpoint as three questions the clinician should ask themselves: if I’m right what do I expect to happen; how will I know if I’m wrong; and what would I do then?^6^ Subsequently, the term ‘safety netting’ has been used to describe a diverse array of activities including: educating patients on symptoms to look out for, explaining the expected time course of illnesses, communicating uncertainty, review at a set time period (follow-up), clinician training, liaison between healthcare professionals, ensuring investigations are reviewed by appropriately trained healthcare professionals and acted on, and other system factors.^3^^,^^4^^,^^7^^,^^8^

As safety-netting activities are so ingrained in clinical practice and recommended in multiple guidelines, randomised controlled trials of managing patients with or without safety netting may be deemed to be ethically inappropriate. Observational studies offer an alternative study design to evaluate safety-netting practices and potential effects on patient outcomes, but currently there are no tools available to systematically assess clinician safety-netting communication behaviours.

Though there have been some quantitative evaluations of safety-netting practices,^8^^,^^9^ many research studies in primary care have been qualitative in nature.^3^^,^^10^^–^12 Quantitative studies based on review of medical notes are limited by the accuracy of the information recorded and qualitative research suggests that documentation of safety netting is poor.^10^ Moreover, previous evaluation of video-recorded primary care consultations has demonstrated that not all problems discussed are documented in the medical notes.^13^ Coding tools, independently applied to observed or recorded clinician–patient interaction, enable the quantification of communication in healthcare encounters.^14^^,^^15^ The assessment of real consultations in this way allows for independent evaluation of complex interactions between clinicians and patients (or carers).^16^

The aim of this study was to develop an interaction coding tool that could be used to quantify when and how ‘safety-netting advice’, see definition in Box 1, is delivered during healthcare encounters, how patients immediately respond to the advice, and what is documented in the medical notes.

**Table:** How this fits in

Recommendations to incorporate safety netting into clinical practice are widespread, but there is a lack of empirical evidence on the extent to which healthcare providers give any safety netting advice and what effects this may have on patient care and safety. Previous research has described the key components that safety netting advice should include, but no coding tools exist to capture which components are enacted in practice, for which problems, and how patients respond to such advice. This article describes the development and testing of a coding tool that can be used to systematically record patient–clinician safety netting communication behaviours.

**Box 1.** Safety-netting advice definition and exclusion criteria

**Safety-netting advice** *‘Information shared with a patient or their carer designed to help them identify the need to seek further medical help if their condition fails to improve, changes, or if they have concerns about their health.’* Adapted from Roland *et al* 2014.^17^	

**Exclusion criteria**	
**Planned follow-up**	Back pain: *‘Then, you need an appointment with me in a couple of weeks’ time.’*
**Contingent on investigation result**	Suspected vitamin D deficiency: *‘If it is low, we will need to restart your vitamin D.’*
**Contingent self-care**	Fungal rash: *‘Then, if you’ve got some left over, keep it in the bedside cabinet, and if it comes back you can just use it again.’*
**Delayed prescriptions**	Indigestion pain and prescription for proton pump inhibitor: *‘And then if, despite that, you’ve still got symptoms, cash this in, probably tomorrow, if you’re still in trouble, OK? ’*
**Changes mind about treatment already offered**	Offering exercise on prescription: *‘If you decide you want to do that, you let me know.’*
**Contingent administration**	New medication: *‘If you wanted them to be put over on to repeat let me know and I can add it to your repeats.’*

## METHOD

### Defining safety-netting advice

After identifying multiple definitions of ‘safety netting’ in the published literature^2^^,^^10^^,^^17^^–^21 and reviewing common themes, the authors chose to separate the generic term ‘safety netting’ from ‘safety-netting advice’.^2^ Roland and colleagues’ definition of safety netting^17^ was adapted to explicitly include the importance of reviewing symptoms if they persist, which has been described as a key element of safety-netting advice in general practice.^22^ In this study safety-netting advice was defined as:

‘Information shared with a patient or their carer designed to help them identify the need to seek further medical help if their condition fails to improve, changes, or if they have concerns about their health.’

Safety-netting advice was distinguished from follow-up: to qualify as safety-netting advice, it had to include contingency planning (predominantly, *‘if x happens then do y’*), whereas follow-up was identified as a non-contingent review or investigation of a problem. During the pilot study, the authors encountered numerous other contingency plans that did not meet their definition of safety-netting advice and listed these under the exclusion criteria with examples for coders (Box 1). Any problems incidentally raised for third parties, for example, child of patient, were also excluded owing to the restrictions set out in the original database collection.^23^

Initially the authors considered including limited prognostic statements, for example, *‘this should get better in 1 week ’,* with the coding tool as a form of safety-netting advice, but on final discussion with the research team and the patient group the authors decided to treat this as a ‘contextual’ code, along with existence of any diagnostic uncertainty, *‘I’m not sure what this is’,* and planned follow-up.

### Data

Consultation recordings used in the development and evaluation of the tool were obtained from the ‘One in a Million’ Primary Care Consultation Archive,^24^ collected during 2014–2015, full details of which are reported elsewhere.^23^ There were 318 unselected adult consultations (300 video, 17 audio-only, one transcript-only) available with consent for use in this project involving 23 different GPs based in 12 GP practices in the West of England. The content of the consultations had previously been transcribed and coded into patient ‘problems’ — defined as the answer to the question *‘What is wrong? ’* — using the Complex Consultations Tool.^25^ The archive also contains linked data in the form of GP and patient demographic information and pre- and post-visit questionnaires.^23^

### Codebook development

In 2016 the authors searched major databases including: EMBASE, MEDLINE, CINAHL, Cochrane Library, Web of Science Core Collection, Scopus, PubMed, and PubMed Central for the term ‘safety-netting’ (which also returns hits for ‘safety netting’) and ‘safety net advice’. A literature review was conducted by the first author, along with a search of clinical guidelines and all articles citing the seminal work by Almond and colleagues.^7^ The authors also drew on existing codes for safety-netting communication behaviours developed in the ‘Understanding the causes of miscommunication in primary care consultations for children with acute cough’ (UnPAC) study of paediatric primary care consultations.^26^ Development work using 93 consultations from the archive obtained by a random sample stratified by GP was conducted to further develop and test the codebook. Consultations containing safety-netting advice were independently assessed by two coders, new codes were generated, and existing codes refined. An iterative approach of coding, discussion, and refinement of the codebook with further examples added to illustrate each code was used.

Five members of the public were recruited to advise on further refinements that would be important to include from a patient’s perspective. Participants were recruited from a list of people who had agreed to be contacted and were reimbursed for their travel and time. Once both coders were satisfied with the contents of the codebook, formal assessment of inter-rater reliability (IRR) of the safety-netting codes was initiated.

### Presence or absence of safety-netting advice

Two coders independently reviewed a random sample of 10% (32/318) of the consultations for the presence or absence of safety-netting advice using both transcripts and consultation recordings. Each coder recorded whether they thought safety-netting advice had been provided for each problem raised in the consultation, recorded the line number where the safety-netting advice started, and highlighted the relevant part of the transcript. Where there was disagreement between coders, a third member of the research team was consulted until a group consensus was met. One coder, the first author, then screened the rest of the database.

### Application of coding tool to a sample of consultations

All consultations or problems identified as including safety-netting advice from this stage of the process were then deemed eligible for coding. An a priori overall target of 85% inter-rater agreement was set. Again, both transcripts and consultation recordings were used together to facilitate accurate coding. Data on safety-netting using the new tool were collected using Microsoft Excel. After coding was complete, data were imported into Stata (version 15.1) for analysis.

### Statistical analysis

Both percentage agreements and Cohen’s *κ*, weighted for partial agreements, were used to assess IRR for categorical data.^27^ Full weightings with explanations for the codes are given in Table 1. To assess IRR for continuous data it is preferable to calculate an intra-class correlation coefficient (ICC) but a quadratically-weighted *κ* is also an accepted method and has been shown to be equivalent to ICC under certain conditions.^28^^,^^29^ A two-way mixed-effects ICC was calculated for the absolute agreement between coders and reported individual ICCs. A mixed-effects model was used because coders were not randomly sampled from a population of potential coders (though ICC estimates for mixed and random models are identical, this notation is only important for interpretation of the ICC estimates).^30^ A mean average percentage agreement and *κ* score were reported for the final coding tool. Both a quadratically weighted *κ* and an ICC were reported for the one code with continuous data.

**Table 1.** Inter-rater reliability scores for final safety-netting tool^a^

**Code**	**Variables**	**Agreement, % (weighted)**	***κ* (ICC)**
**Safety-netting contextual codes**			
Diagnostic uncertainty	No, yes, n/a	80	0.62
Expected time course of illness	No, yes, n/a	83	0.66
Follow-up	None, investigation only, practice, same GP, other, multiple	74 (83)^b^	0.77^b^
Follow-up documentation^c^	No, yes, CBD, n/a	100	1

**Safety-netting advice codes**			
Applicable to problem, treatment or management plan, or both	Problem, treatment or management plan, both	88 (92)^d^	0.75^d^
Stage of the consultation	Establishing reason, gathering information, delivering diagnosis, treatment planning, closing, unclear	82	0.67
Initiation	Clinician, patient	96	0
Format	Conditional plus course of action, conditional warning only^e^	98	0.79
Strength of endorsement	Weaker, neutral, stronger	94	0.87
Conditions/symptoms, *n*	1–20	84 (99) ^f^	0.85^f^ (0.86)
Generic or specific advice	Generic, specific	80	0.61
Action advised^g^	None (conditional only), other in-hours, practice, same HCP, OOH, 999	88	0.78
Timescale of action^g^	Not specified, fixed, immediate	92	0.80
Focus of action	No action, clinician focused, patient focused, both	84 (89)^h^	0.79^h^
Patient response	No response, resists, nods only, acknowledgement or accepts	80	0.55
Patient questions	No, yes	97	0.65
Written information	Verbal only, verbal and written, unclear	94	0
Documentation^c^	No, yes, CBD	85	0.71

**Total mean average**		88 (90)	0.66

a A total of 51 discrete episodes of safety-netting advice for 35 problems in 24 consultations were recorded.

b Weight: 0.5 (half correct) if multiple matched to any other code other than none.

c Inter-rater reliability not assessed when n/a as no follow-up or safety-netting advice, or when no medical records available.

d Weight: 0.5 when both matched with either problem or treatment.

e One variable dropped, limited prognostic statement only.

f Quadratic weighting.

g From a repeated cycle of coding tool analysis based on 25 episodes of safety-netting advice across 13 problems from 10 consultations.

h Weight: 0.5 when both matched with either clinician- or patient-focused action. CBD = cannot be determined. HCP = healthcare professional. ICC = intra-class correlation coefficient. n/a = not applicable. OOH = out of hours.

## RESULTS

### Sample characteristics

The unstratified random sample of 32/318 patients contained consultations from 19/23 GPs (9 male, 10 female) in the archive, working across 12 practices, with a range of one to three consultations from the same GP. All GPs included in the screening were of a self-reported white ethnic group with an age range of 32–62 years and a mean average age of 47 years. There were 15 male and 17 female participants with an age range of 20–83 years, with a mean average age of 48 years. Of the patients, 27 described themselves being white and four reported belonging to another ethnic group; see Table 2 for patient characteristics.

**Table 2.** Patient characteristics^a^

**Characteristics**	**SNA screening, *n*(%) (*N*= 32)**	**Full tool application, *n* (%) (*N*= 24)**
**Sex**		
Male	15 (46.9)	10 (41.7)
Female	17 (53.1)	14 (58.3)

**Age, years**		
18–34	8 (25.0)	7 (29.2)
35–49	8 (25.0)	5 (20.8)
50–64	7 (21.9)	6 (25.0)
≥65	6 (18.8)	3 (12.5)
Not reported	3 (9.4)	3 (12.5)

**Ethnic group**		
White	27 (84.4)	19 (79.2)
Other	4 (12.5)	4 (16.7)
Not reported	1 (3.1)	1 (4.2)

**IMD quintile**		
1 (least deprived)	10 (31.3)	5 (20.8)
2	7 (21.9)	6 (25.0)
3	3 (9.4)	3 (12.5)
4	1 (3.1)	1 (4.2)
5 (most deprived)	11 (34.4)	9 (37.5)

a The full coding tool was applied to all consultations from the screening process that contained safety-netting advice. IMD = Index of Multiple Deprivation. SNA = safety-netting advice.

### Tool components

The final tool compromised four main types of codes: administrative codes; safety-netting contextual codes; safety-netting advice codes; and an additional optional set of problem contextual codes. Administrative codes recorded assigned study identification number, how many problems were raised during consultation, and the type of problem using the International Classification of Primary Care (ICPC-2) classification.^31^ These administrative codes were based on information from the original ‘One in a Million’ study and not included in the IRR testing.

Contextual codes recorded elements that have been described as key features of the broader term ‘safety netting’ such as the communication of planned follow-up, diagnostic uncertainty, and the expected time course of a problem,^7^ but these features would not be coded as ‘safety-netting advice’ as standalone statements. As this study aimed to capture all statements of uncertainty around the diagnosis, the authors opted to include both direct, *‘I’m not sure what the diagnosis is’*, and indirect, *‘I think it’s x ’*, statements of uncertainty.^32^

Codes that evaluated the specific details of the safety-netting advice formed the bulk of the coding tool. An example of how these codes are assigned to an extract of a GP giving safety-netting advice is provided in Box 2. As multiple consultation models teach that safety netting is delivered towards the end/during the closing-down phase of a consultation,^6^^,^^33^ the authors wanted to be able to capture when in the consultations GPs were actually giving safety-netting advice and who was initiating the advice-giving sequence. The phases of the consultation based on the original classification described by Byrne and Long (Phase II to Phase VI)^34^ were recorded but links to the corresponding phases for the Calgary–Cambridge model were also provided.^33^

**Box 2.** Coding example

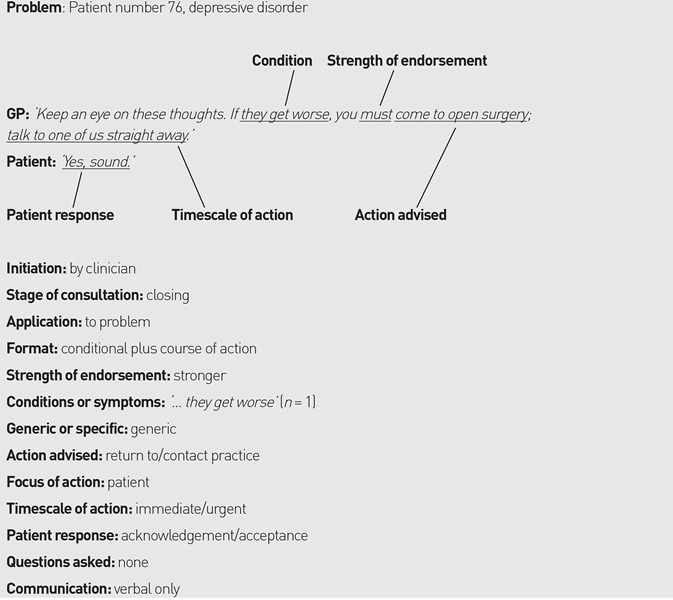

During development work the authors also observed doctors giving safety-netting advice that only applied to their treatment or management plan, therefore a code to differentiate between problem and treatment safety-netting advice was generated, as demonstrated in Box 3.

**Box 3.** Examples of safety-netting advice for a problem, for a treatment or management plan for the problem, or both

**Problem**	*‘So, reassured about this, but come back to us if it seems to be changing.’*
**Treatment/management plan**	*‘Antiinflammatories are really good at pain thinning, but they’re bad at irritating the lining of the stomach. And in the worst case it can cause an ulcer and bleeding. So, if you’re getting indigestion pains, coughing up blood, or your stool is very dark and black and sticky, you must stop the naproxen and come and see me straight away.’*
**Both**	*‘Yes, well, any problems, come back.’* *‘And of course, if things are getting worse rather than better in the meanwhile, or any problems with the antibiotics, we’ll see you before.’*

The number of conditions or symptoms the doctor had warned the patient to look out for, the action they should take if those symptoms developed, and how quickly they needed to take such action was recorded. Regarding the course of action recommended, the codes included three different options: patient-focused action, *‘****you*** *must come back’*; doctor-focused action, *‘****I’d*** *like to have another look at it ’*; and both doctor- and patient-focused action, *‘****you*** *must come back so* ***I*** *can have another look at it ’.*

In addition, a code to separate generic from specific advice was generated, full criteria for which are described in detail in the codebook (Supplementary Table 1). Briefly, generic advice could potentially apply to multiple problems or management plans, for example, *‘any problems’*, *‘issues’*, *‘it gets worse’*, or asking the patient to return if their condition did not get better but without giving a specific time frame; whereas specific advice included new symptoms such as *‘chest pain’*, *‘cough up any blood’*, or asking them to return if their symptoms persisted but included a time frame *‘If it’s not better in* ***2 weeks*** *then come back ’*.

Finally, how the patient responded to the advice, if the patient asked any questions about the advice, if the doctor gave any written safety-netting advice, and whether the doctor documented that they had given safety-netting advice in the medical records was assessed.

Multiple other contextual codes were included to help identify which types of problems were associated with higher or lower rates of safety-netting advice, such as the nature of the problem, for example, ‘acute’ or ‘chronic’. As these codes were not directly related to ‘safety netting’ the authors opted to include these in an optional section.

### Patient and public involvement group

The patient and public involvement (PPI) group review of the coding tool led to the development of two further codes: does the doctor check the patient has understood the advice? And does the patient ask any questions about the advice? The group also helped with the refinement of one existing code: action advised as part of the safety netting advice to include an option to seek help from another in-hours provider such as a pharmacist.

### Collapsed codes and repeat evaluation

Inter-rater testing demonstrated that some variables were too difficult for coders to reliably distinguish between. For example, when assessing how patients responded, coders found it difficult to differentiate between codes for acknowledgements *‘yeah’*, positive assessments *‘great, fine ’*, and acceptance *‘OK, all right, sure ’*. These codes were subsequently collapsed into a single ‘acknowledgement or acceptance’ code. Two codes (the action advised and the timescale of action) that were deemed to have performed substandardly but were judged to be essential to retain underwent further refinement and were evaluated in 10 further randomly selected consultations that contained safety-netting advice (13 problems, 25 discrete episodes of safety-netting advice) using the relevant parts of the written transcripts only. The authors also wanted to differentiate whether coders thought the safety-netting advice and follow-up plans had been fully or only partially documented in the patient’s medical notes and if diagnostic uncertainty and the expected time courses of illness were delivered with the safety-netting advice or at a separate part of the consultation, but IRR scores demonstrated that in its current format coders could not reliably discriminate to this level of detail.

### Adjustments and dropped codes

Three codes were removed from the tool as the coders struggled to reliably differentiate between the different variables. A list of all the dropped codes and their IRR scores are shown in Supplementary Table 2. Two contextual codes, which both had substantial IRR score — ‘is this the first presentation with this problem to healthcare professional?’ (agreement 89%, *κ* = 0.71) and ‘is a diagnosis given’ (agreement 74%, *κ* = 0.62) — were deemed non-essential to the coding tool on final review and therefore moved to the optional section of the coding tool. This decision was made primarily to reduce the time taken to complete the coding tool.

### Inter-rater reliability scores

At the consultation level, coders agreed on the presence or absence of safety-netting advice for 32/32 consultations (100% *κ* = 1.0). At the problem level, coders agreed on the presence or absence of safety-netting advice for 49/55 problems (89% *κ* = 0.77). The ICC for the number of separate times safety-netting advice was discussed in each consultation and for each medical problem was 0.88 and 0.73 respectively. A contributing factor towards the lower ICC for safety-netting advice per medical problem was if generic safety-netting advice, for example, *‘any problems let me know’*, was not listed under all the problems it could have applied to.

Incidents where one coder missed an episode of safety-netting advice but incorrectly labelled a non-safety-netting contingency plan (exclusion criteria, Box 1) occurred once for coder 1 and three times for coder 2. This only positively affected the IRR results for the presence or absence of safety-netting advice for one consultation and one medical problem. Because agreement scores do not differentiate between correct agreements and false agreements, the authors also reported how many of the 51 discrete episodes of safety-netting advice each coder correctly identified and any false positives. Full details of correct and false positive identification of safety-netting advice by coders are shown in Supplementary Table 3. Coder 1 correctly identified 48/51 (94.1%) episodes of safety-netting advice, whereas coder 2 correctly identified 45/51 (88.2%) of episodes.

The review process identified 51 separate episodes where the GPs gave safety-netting advice for 35 problems in 24 consultations. Table 1 demonstrates the IRR scores assessed in the coding tool. One code ‘does the safety-netting advice apply to this problem or multiple problems?’ was wholly dependent on the screening for the presence or absence of safety-netting advice for each problem, therefore IRR was not reassessed. The mean average unweighted percentage agreement was 88% (90% weighted) and mean average *κ* score was 0.66 for the final tool.

### Final tool

The final codebook and coding tool are included as Supplementary Tables 1 and 4. As the codebook is publicly available online, the exact extracts from the consultations that were utilised in the development of the codebook have been replaced with example data.

## DISCUSSION

### Summary

To the best of the authors’ knowledge, the development and initial evaluation of the first tool specifically designed to capture when and how safety-netting advice is delivered and received in healthcare encounters is described here. The tool allows for quantification of all the different components of safety-netting advice including: whether the clinician or patient initiate the advice; the stage of the consultation at which the advice is discussed; the formatting of the advice; the strength of the advice endorsement; the number and type of conditions or symptoms the patients are informed to look out for; whether the advice is generic or specific; how the patient should seek further help and how quickly they need to act; how patients respond to the advice; whether patients ask further questions about the advice; if any written information is given; and if the clinicians document any safety-netting advice in the medical notes. The tool also includes codes designed to capture contextual data for each problem raised during the consultations, such as the communication of any diagnostic uncertainty, the expected time course of the illness, and whether any follow-up is arranged for the problem.

### Strengths and limitations

In the face of uncertainty around the reach of the term ‘safety netting’, a clear definition of ‘safety-netting advice’ was generated based on the published literature and a robust set of inclusion and exclusion criteria was created that was grounded in the published literature, clinical experience, and, perhaps most importantly, from watching multiple real-life consultations.

IRR testing between two coders demonstrated the tool could be reliably used to evaluate safety-netting advice in GP consultations with an overall percentage agreement of 88% (unweighted), which exceeded the pre-set target of 85% for this study. The mean average *κ* score of 0.66 is deemed ‘substantial’ agreement by Landis and Koch, and falls into the second highest category of agreement levels.^35^

Only one episode was not adequately explained by the categories available in the format section of the codebook. This example was omitted from the IRR scoring and the codebook updated with this minor alteration. Though it is possible that there might be other cases that could be considered safety-netting advice that may not fit the codebook, the tool has been extensively tested and applied to a total of 390 episodes.^36^

When assessing IRR, weighted *κ* scores were used with caution owing to their inherent subjectivity but potentially could have been utilised more. For example, the authors asked coders to differentiate if the action advised in the safety-netting advice was to return to the practice or specifically the same GP. These actions are very similar, and, potentially, it would be too stringent to treat them as unweighted codes, which may underestimate the true IRR of the tool.

Though *κ* scores are a widely accepted method of assessing IRR they are not without issues. First, two codes (who initiates the advice; and is written advice given) returned *κ* scores of 0 yet had high percentage agreements (96% and 94% respectively). This is because of the very low incidence of patient-initiated safety-netting advice and written advice, which creates a high level of expected agreement in the *κ* statistic calculation. This penalisation is recognised as part of the *κ* statistic ‘paradox’ and therefore may underestimate the IRR of the coding tool.^37^ Both weighted and unweighted percentage agreement scores have been provided for this reason. Second, *κ* scores are also influenced by the number of variable categories available and therefore the individual *κ* scores for each code best represent the IRR rather than the overall mean average *κ*.^38^^,^^39^

In an ideal coding tool all codes should be independent of one another to avoid double penalisation or reward of a single decision, which was not always possible. For example, the documentation of follow-up is dependent on whether the coder thought any follow-up was present in the first instance. To avoid double penalisation or reward only IRR of this code was assessed when both coders agreed there was some form of follow-up. Fortunately, there were no cases where coders disagreed that there were at least some follow-up plans in place and all disagreements originated from the type of follow-up code. Furthermore, to avoid over-inflating IRR scores for documentation codes, agreement scores were not included if no medical notes were available.

Some codes that returned poor IRR scores had to be collapsed down into broader categories or removed. Two codes that were deemed essential had to undergo a further round of testing after additional refinement. Other limitations included the fact that only coder ratings by two coders were used in the formal assessment of IRR, both of whom were involved in the development of the tool. However, by creating a detailed codebook to accompany the tool, the authors have included specific examples and clear explanations to aid assignment of codes for future coders. Furthermore, when multiple actions were included in the safety-netting advice, the authors chose to code the highest ‘action’ variable recommended. For example, if a GP recommended that a patient return to them for help or if it was out-of-hours (OOH) to ring 111, the action would be coded as ‘contact OOH services’. Future editions of the coding tool could potentially map different symptoms listed by the GP onto separate actions, but this may make the coding tool more labour intensive.

### Comparison with existing literature

Although there are numerous other coding tools that assess other specific parts of the consultation, this study is the first to describe a tool specifically designed to quantify the delivery and receipt of safety-netting advice.^14^^,^^25^ A comparable iterative method of code generation and refinement was used, followed by rigorous formal IRR testing using a well-recognised method of agreement grading.^35^ One other study in Danish primary care has utilised traditional conversation analysis (CA) methods to evaluate safety-netting communication behaviours^40^ and, though the tool in the present study has some foundations built in CA methods, it is designed to require much less training to apply than full CA.

### Implications for research and practice

The presented tool will allow for the systematic assessment of safety-netting communication behaviours in video- and audio-recorded primary care consultations.^36^ This will permit further evaluation of prior research findings, for example, that safety-netting advice is often not documented in the medical notes and tends to be vague^10^^,^^41^

The present findings suggest that this tool, in its current format, cannot be used to reliably determine if the GP checked patient understanding of the safety-netting advice (low IRR). It may only be possible to assess this by means of a patient-completed questionnaire after the consultation. This is likely to provide a more accurate assessment of patient understanding, as patients may be too embarrassed to admit that they have not understood a doctor’s advice when asked directly in a consultation.^42^ Minor adaptations, such as reducing the number of codes, may be required for use in live consultations where immediate feedback is required, as the current tool was tested on recorded consultations where coders had the ability to replay sections of the consultation.

In summary, the authors have developed a tool that can be used to systematically evaluate clinician safety-netting communication behaviours, but further research is required to determine how patients interpret different forms of safety-netting advice and the effect they might have on clinical outcomes.
